# Psychoeducational family intervention for bipolar I disorder: medium and long-term efficacy

**DOI:** 10.1192/j.eurpsy.2022.1936

**Published:** 2022-09-01

**Authors:** L. Marone, S. Marzolo, M. Raia, V. Giallonardo, V. Del Vecchio, G. Sampogna, M. Luciano, A. Fiorillo

**Affiliations:** Università degli Studi della Campania Luigi Vanvitelli, Psychiatry, Napoli, Italy

**Keywords:** Bipolar I Disorder, psychoeducation, Family burden, coping

## Abstract

**Introduction:**

Bipolar disorder (BD) is associated to high personal and social burden, impaired social functioning and high levels of disability. Recent studies have showed that relapse rates are significantly reduced in those patients whose families receive psychoeducational interventions. Even though most of available evidences are related to the short-term efficacy of psychoeducational family interventions (PFI). No evidence is available on medium and long-term efficacy.

**Objectives:**

This study aims to assess the efficacy after one and five years of PFI in BD in terms of: 1) improvement of patients’ symptoms and global functioning; 2) improvement of relatives’ objective and subjective burden and coping strategies.

**Methods:**

A multicenter, controlled, outpatient trial has been conducted in BD patients and their key relatives, recruited in 11 Italian mental health centers. Patient’s clinical status, social and personal functioning, burden of illness, and relative’s burden and coping strategies were assessed with specific instruments at baseline, after 1 year and after 5 years.

**Results:**

137 families were recruited, 70 allocated to the experimental intervention. After one year, an increasing positive effect on patients’ clinical status, global functioning and objective and subjective burden was found. Moreover, were observed a reduced number of relapses and of hospitalizations after five years, compared to the control group. A reduction in the levels of family burden and an improvement of their coping strategies were also observed.

**Conclusions:**

Positive effects of the experimental intervention persist over the mid and long-term period. PFI should be provided in mental health centres to patients with BD and their relatives.

**Disclosure:**

No significant relationships.

